# Preanalytical Quality Evaluation of Low-Volume Citrate Evacuated Blood Collection Tubes—Anticoagulant Solution Volume Accuracy, pH, and Anionic–Cationic Composition

**DOI:** 10.3390/molecules31091516

**Published:** 2026-05-02

**Authors:** Nataša Gros, Zala Hriberšek

**Affiliations:** Faculty of Chemistry and Chemical Technology, University of Ljubljana, Večna pot 113, SI1000 Ljubljana, Slovenia; zh6804@student.uni-lj.si

**Keywords:** blood collection tubes, citrate anticoagulant concentration, ion exchange chromatography, anionic contaminants, cationic contaminants, dye-dilution method, anticoagulant volume accuracy

## Abstract

Blood collection tubes are widely used medical devices. Inaccurate citrate anticoagulant concentration can influence the results of coagulation tests. The producer’s expertise and responsibility are considered the quality safeguards. However, the tubes undergo changes during their lifecycle, partly due to storage conditions, and the end user or a third party has no comprehensive insight. A methodology is necessary to reveal the tube’s inherent characteristics. We provide insight into the anionic–cationic composition and pH of anticoagulant solutions in commercial tubes using high-performance ion exchange chromatography on a purified water model, making the anticoagulant volume accuracy assessment possible through a direct dye-dilution method. The results revealed differences between the tubes of two producers, Greiner BIO-ONE (A and A(nr)) and BD (C). Tube C has the most accurate anticoagulant amount. Both brands contain buffered citrate. The method of buffer preparation is not a source of interferant for the spectrometric method of the tubes’ quality evaluation. Acetate, formate, chloride, nitrite, sulfate, oxalate, bromide, and nitrate impurities were determined in anticoagulant solutions, all in tube A and some in the others. Tubes C exhibit the highest contamination with cations.

## 1. Introduction

For a blood collection tube to operate correctly, several parameters should be compliant. Since all its components can contribute to errors, a tube as a medical device can be considered an undermined preanalytical variable [1]. The tubes containing anticoagulant in a dry form or as a solution of a negligibly small volume have the correct anticoagulant level at the specimen collection if the anticoagulant amount (*n*_AC_) and draw volume (*V*_Draw_) are accurate. Such examples are K_3_EDTA or K_2_EDTA tubes. Citrate anticoagulant tubes are more complex, since they contain the anticoagulant solution, which is assumed to dilute ten-fold at the specimen collection to reach a concentration of 10.9 mmol/L. To achieve the goal, in addition to accurate *V*_Draw_, the anticoagulant should be of accurate concentration (*c*_AC_) and volume (*V*_AC_).

It must be considered that during the tube’s lifecycle the internal under-pressure fades, and water loss can accompany it. Storage conditions contribute to this problem. Even though the anticoagulant amount remains constant, the anticoagulant concentration and volume might change, consequently affecting the blood specimen anticoagulant concentration level.

Inaccurate citrate anticoagulant concentration can influence the results of coagulation tests. An early study, by delivering different volumes of a syringe-drawn blood sample into Vacutainer tubes, estimated that 30% under-fill and 40% under-fill, respectively, are acceptable for activated partial thromboplastin time (APTT) and prothrombin time (PT) assays, if 3.2% citrate tubes were used [2]. Changes in anticoagulant volume and concentration were not considered. It was further confirmed that samples with elevated hematocrit require citrate anticoagulant level adjustment for accurate APTT and PT determination [3]. The low hematocrit effect on coagulation tests [4] is less frequently mentioned. Excessive citrate concentration in blood, which is characteristic of some medical conditions, can lead to misdiagnosis [5]. Preventive measures based on the ISO 15189:2022 standard have to be implemented for PT and APTT assays [6].

Several different materials and additives are involved in tube production, and their impacts on blood tests were widely studied [7]. For citrate tubes, magnesium leaching from a stopper was recognized early as an impurity that affected the PT ratio [8]. Other magnesium-related studies followed [9,10,11]. Atomic absorption spectrometry is a convenient method for the determination of magnesium and other alkaline earth metals and alkali metals. To the best of our knowledge, no studies report anionic citrate contaminants.

The tubes are presumed to conform with legislation during their shelf life, and not to release unknown additives or contaminants [12]. The responsibility is on the side of the producer. Regarding the quality control of a tube as a product, standards only suggest a pre-production titrimetric method for the evaluation of anticoagulant solution, and thus provide very little guidance [13]. To the best of our knowledge, a comprehensive pre-analytical methodology of evaluating *V*_AC_, *c*_AC_, and *n*_AC_ in a tube using a water model was not reported in the scientific literature by any other research group, and the methodology we previously developed was not complete or easy to implement.

Consequently, the end user can only rely on the declared characteristics and comparability of behavior on blood samples [14], for which all steps of the total laboratory testing process are involved [15,16,17]. The judgement of the tube interchangeability is statistically based. Even though it is an important and strictly necessary quality control measure, it does not expose the inherent tube characteristics. Consequently, cause-and-effect-based considerations are not possible.

An alternative insight can serve official bodies and end users. On the other hand, independent third-party research of the tubes’ characteristics and behavior provides feedback and gives additional tools to the producers and prospective producers. During product development, the latter can avoid venipuncture until it becomes justifiable.

A methodology to test tube characteristics using a water model, namely *V*_AC_, *n*_AC_, and *c*_AC_, is supposed to be low cost, easy to implement, and involve instruments that are common and not too sophisticated. In a water model it was not possible to apply conductometry, which worked well with K_2_EDTA and K_3_EDTA tubes, since the electric conductivity of buffered or unbuffered citrate solutions differs.

**Figure 1:**
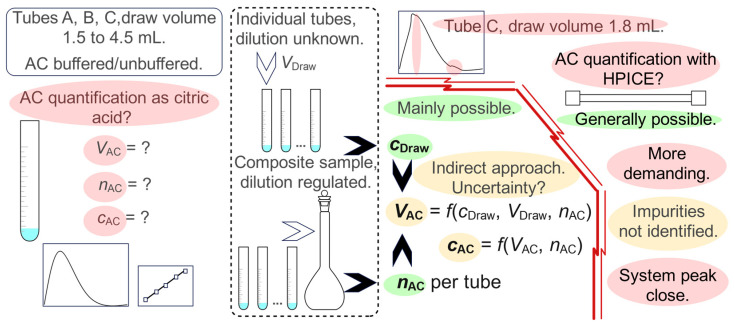
Challenges, resolved issues, and partially resolved issues (red, green, yellow) of the previous attempts at citrate anticoagulant (AC) tubes evaluation. Symbols *V*, *n*, and *c* stand for volume, amount of substance, and amount concentration, respectively. Left of the gap symbol, a spectrometric study [18] is presented, combining two different approaches to tube evaluation (dashed frames), to resolve the issue of unknown anticoagulant volumes. Right of the gap symbol, a comparative study [19]), involving ion exclusion chromatography (HPICE) with an additional objective of identifying the spectral interferent, is outlined.

Previous attempts to develop a citrate tube methodology are summarized in Figure 1. The shades of red, yellow, and green, respectively, indicate a research question or further challenge, a partially resolved issue or indirect determination, and a resolved issue or direct determination. Citrate anticoagulant quantification using citric acid proved mainly possible with molecular absorption spectrometry in the ultraviolet range (UV) [18]. By combining two approaches performed in a water model, specifically citrate concentration determination in individual tubes at *V*_Draw_ and determination in a composite sample, it was possible to derive *V*_AC_ and *c*_AC_ (Figure 1, left of the gap symbol). Due to unknown *V*_AC,_ determination of *n*_AC_ in individual tubes was not possible, and only the *n*_AC_ per tube estimation was deduced from the composite sample. The tubes of the three producers were tested, namely the Vacuette^®^, Greiner BIO-ONE; Vacutube, LT Burnik d.o.o.; and BD Vacutainer^®^ tubes, marked A, B, and C, respectively. The nominal draw volumes ranged from 1.8 to 4.5 mL. In the 1.8 mL tube of brand C, an unknown additive or impurity influenced the citrate absorption spectrum shape, consequently limiting its evaluation with spectrometry.

The method of citrate anticoagulant determination with ion exclusion chromatography (HPICE) was developed for comparison and interferent identification [19]. It was applied to the 1.8 mL tubes of all three producers that proved the least compliant. The method was confirmed to be generally applicable, but it has disadvantages and the interferent was not identified (Figure 1, right of the gap symbol).

**Figure 2:**
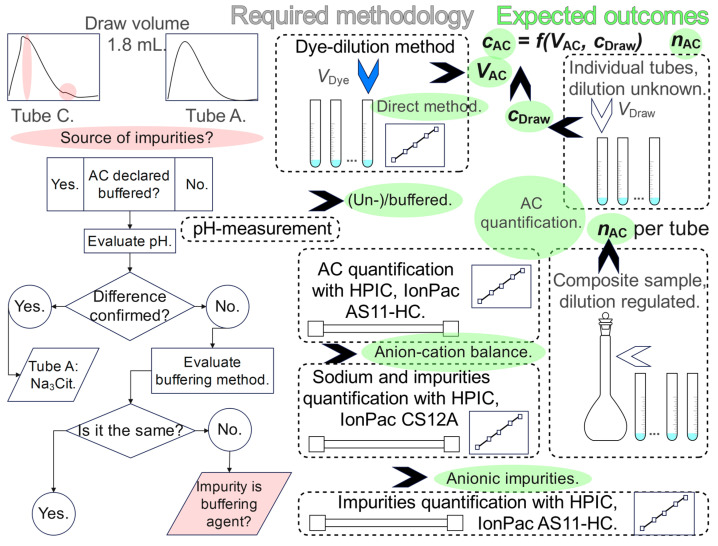
Reasoning, methodology and expected research outcomes (green) for prospective citrate anticoagulant tube evaluation. Left, strategy of evaluation of the interferent origin; dashed frames, methodological approaches; the HPIC abbreviation stands for ion exchange chromatography.

The background reasoning and considerations for upgrading the methodology to reach the goals are outlined in Figure 2. Citrate anticoagulant is either buffered or unbuffered, and opinions regarding clinical implications differ [20,21]. However, methods of preparation of the buffered citrate can vary. Either trisodium citrate and citric acid are combined, or trisodium citrate as a precursor is partially transformed into other equilibrium forms by the addition of an acidic substance. Citrate of the 1.8 mL tube C, with a distortion in the spectrum shape, was declared buffered, but none of the others. If a UV-light-absorbing substance was used, it could have affected the citrate absorption spectrum. The two methods of buffer preparation result in a different sodium to citrate proportion.

Based on Figure 2, the objectives of this work can be summarized as follows. (1) A direct method of *V*_AC_ determination should replace the indirect method to enable the determination of *c*_AC_; this will be more reliable and allow quantification of the *n*_AC_ in individual tubes. A dye-dilution method is supposed to be the right choice for *V*_AC_ determination. (2) Reliable determination of anion–cation balance associated with metrologically traceable pH measurement is necessary to reveal a buffer-preparation method of tube C. The method, which was previously used only to determine cationic impurities with ion exchange chromatography (HPIC), should be upgraded to enable reliable determination of sodium as the major cation. (3) Ion exchange chromatography should replace ion exclusion chromatography to enable the reliable determination of citrate ions and the identification of anionic impurities, which were previously not able to be identified with HPICE. Justification of some methodological details, already presented in Figure 2, follows.

In this study, we limit ourselves to the low-volume, 1.8 mL draw volume citrate tubes of producers A and C, for which we previously observed influences on absorption spectra, to a greater extent in the latter than in the former. The low-volume tubes receive more attention in clinical evaluations [22,23,24,25] since they are more frequently used.

The dye-dilution technique emerged in the early 1950s and enabled cardiac output measurements [26]. It became widely used in medicine at the beginning of the 1960s [27] and remains a diagnostic tool [28]. Revaluation of the Evans blue dye-dilution method of plasma volume measurement remains broadly recognized [29]. One of the more recent applications not related to medicine is dye-dilution-based evaluation of mixing in a microfluidic system [30].

Calibration of micropipettes of the volumes between 1 and 50 µL by using a solution of nicotinamide as a light-absorbing substance was an early application in discrete systems [31]. With the dye-dilution method, the volumetric accuracy and precision of a positive-displacement microliter diluting device were evaluated [32]. While the metrological institutions recognized the dye-dilution method for flow measurements [33], the development of high-accuracy and precision balances for lower masses caused gravimetry to become the method of choice for volume evaluations in general. The dye-dilution-based volume measurements were later revived either as a dual-dye method [34] or a single-dye approach [35], where high throughput was necessary [36] and usually in association with plate readers. For anticoagulant volume determination, a dye-dilution method development will require an immersive probe with vertical optical geometry to enable in situ measurements.

The ion exclusion chromatographic method involving an IonPac ICE-AS1 column enabled citrate anticoagulant quantification, but the citrate peak was unfavorably close to the system peak, and impurities were not detected [19]. With reversed-phase high-performance liquid chromatography (HPLC), citrate can be determined in plasma [37]; nonetheless, for the determination of anion–cation balance, anion exchange chromatography with suppressed conductometric detection better suits the purpose. With the Dionex IonPac^TM^ Fast Anion IIIA column, dedicated to citrate quantification together with inorganic ions, the citrate in urine was determined [38]. The IonPac AS11 column, in addition to citrate determination, enables the determination of other organic acids and several inorganic anions. Diverse isocratic and gradient methods were reported [39,40,41,42,43,44,45]. Two different isocratic methods will need to be developed to enable, on the one hand, the quantification of citrate as a macro-component, and on the other hand, the identification and quantification of impurities.

The main innovations of this paper are:

- A direct determination of anticoagulant solution volume in an evacuated blood collection tube is possible for the first time. The dye-dilution method is transferable to plate readers used in medical laboratories. The checks of anticoagulant volume, as a conformity and lifecycle indicator of the tube, are enabled and easy to carry out.
- By combining metrologically traceable pH measurement with chromatographic methods for the determination of cationic–anionic composition, we made a distinction between different methods of buffer preparation. The methodology is transferable to other areas.

The values of *V*_AC_, *c*_AC_, and *n*_AC_, and the factor of the AC dilution at a draw volume can now be determined in individual tubes of different producers. The method’s performance and fitness for purpose were evaluated. Full validation including a ruggedness test should follow.

## 2. Results

Section 2.1, Section 2.2 and Section 2.3 are dedicated to the development and performance evaluation of chromatographic methods. Similar aspects of the three methods are presented together. Development and performance evaluation of the dye-dilution method follow in Section 2.4. In Section 2.5, the results of tube evaluations are given, namely the pH examination using a water model; the determination of the anticoagulant volume and the amount concentrations of sodium and citrate as dominant ions, determined in parallel in individual tubes; and the determination of the amount concentrations of anionic impurities determined in composite tube samples.

### 2.1. Ion Exchange Chromatography—Optimization of Separations

**Figure 3:**
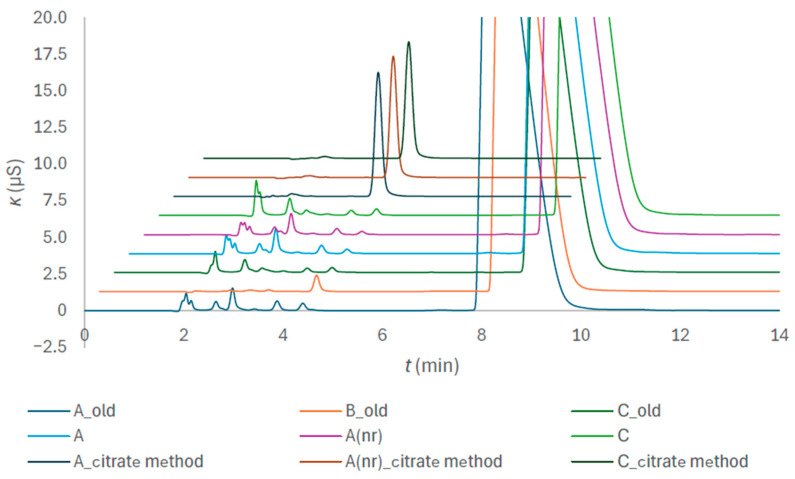
Comparison of chromatograms of the citrate method using KOH of 35 mmol/L (the _citrate method suffix in legend) and method for anionic impurities using KOH of 25 mmol/L on the example of the composite samples of the tubes of the brands A, A(nr), B and C. Composite samples were prepared by transferring anticoagulant solution of several tubes of the same kind into a volumetric flask to ensure a defined dilution.

Two separate isocratic methods had to be developed for anion determination since the requirements for citrate quantification were contradictory to the requirements for the determination of anionic impurities. The eluent strength and sample dilutions differed. Chromatograms of the tubes A, A(nr), C, A_old, B_old, and C_old are presented in Figure 3. The B-brand tubes from the preceding study were included, since in contrast to the tubes A and C, no spectrum-shape distortions were observed, indicating low contamination with species absorbing UV light in HCl medium.

**Figure 4:**
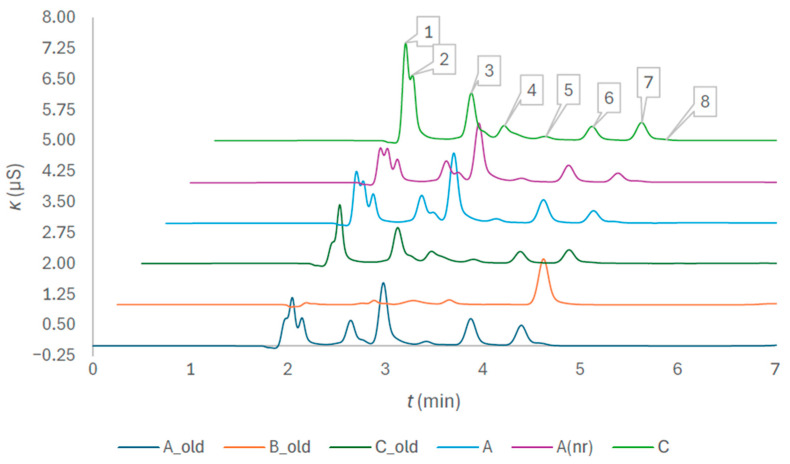
Chromatogram of composite samples prepared from the tubes of different brands; the peak numbers are 1 for acetate, 2 for formate, 3 for chloride, 4 for nitrite, 5 for sulfate, 6 for oxalate, 7 for bromide, and 8 for nitrate.

The suffix nr means non-ridged. More details on the tubes are given in Section 4.1. The suffix _citrate method distinguishes the chromatograms for citrate quantification from the chromatograms of the impurity determination. The two major peaks at approximately 4 and 8.5 min pertain to citrate; the between-method difference is reflected in the peak shape and size. More details on other peaks are given in relation to Figure 4.

The within-day repeatability of retention times, *t*_r_ and peak area were tested. The relative percent standard deviation, *s*_r_(%), appearing in Table 1, is defined by Equation (1) and derived from the sample standard deviation, *s*, and the sample mean, $\bar{x}$.

(1) $$s_{r}(\%)=100\times s/\bar{x}$$

As Table 1 demonstrates, repeatability of fundamental chromatographic parameters is comparable to the that of ion exclusion method for citrate determination [19], but the elution time is shorter, and the citrate peak is better separated from the system water peak (Figure 3). The 95% confidence intervals of retention times ranged between 0.01 and 0.03 min. The 95% confidence intervals of peak area ranged between 0.001 and 0.006 AU.

Figure 4 confirms the presumption that anionic impurities will be revealed by switching from ion exclusion to ion exchange chromatography. The retention times of the peaks that were able to be quantified in anticoagulant solutions of the tubes of different brands are 1.980 for acetate, 2.045 for formate, 2.650 for chloride, 2.972 for nitrite, 3.418 for sulfate, 3.885 for oxalate, 4.392 for bromide, and 4.601 min for nitrate. The amount concentrations are provided at the end of Section 2, together with other tube quality evaluation results.

Different peak profiles are observed for different brands, with the similarities between the older and more recent lots. The anticoagulant solution of brand B proved the least contaminated but had the highest peak of bromide ion of all the tubes, which at the same time was the only one that was clearly expressed. The effect on the absorption spectrum was not observed. The peak profiles of brands A and A(nr) are more complex than the profiles of brand C, and all the peaks were not able to be identified. The highest of all the brands is the peak of nitrite. Acetate, formate, and chloride dominate the peak profiles of tube C. Chloride cannot be considered a spectral interferant. Apart from the bromide peak, other peaks of brand C appear weaker or similarly expressed as those of brands A and A(nr). Since the acetate and formate peaks of tubes C_old and A_old are of a similar height, the difference observed in absorption spectra of the two brands could not have been caused by them. Impurities identified with ion exchange chromatography are probably not the cause of the difference in the two absorption spectra. They might have had some summative effect on the declining right side of both spectra, but they cannot explain the distinct right-side distortion in the spectrum of tube C. The pH measurement and sodium to citrate fraction evaluation will reveal if the distortion effect can be attributed to a citrate anticoagulant buffering agent of tube C.

**Figure 5:**
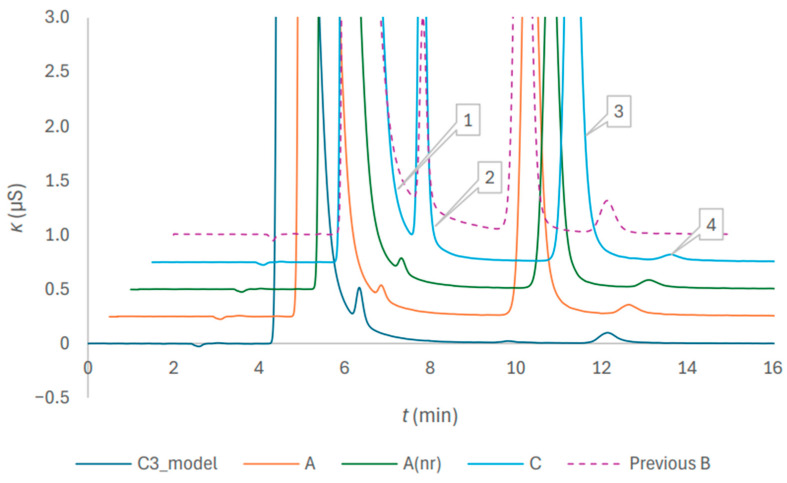
Chromatograms of trisodium citrate model solution and citrate anticoagulant solutions of the blood collection tubes of three brands after the addition of Milli-Q in a volume corresponding to the nominal draw volume of 1.8 mL, followed by a two-fold dilution. The peak numbers are 1 for sodium, 2 for potassium, 3 for magnesium and 4 for calcium; the eluent was 20 mmol/l methanesulfonic acid. The trace Previous B obtained with the 22 mmol/L eluent is included for comparison.

Quantification of sodium as the major cation was previously out of the scope, since the focus was on the cationic impurities. However, for anion–cation balance judgement, high accuracy of the determination of sodium amount concentration is essential. To achieve this goal, improvement in separation was required. Figure 5 demonstrates the enhancement of the quality of separation for the determination of sodium, potassium, magnesium, and calcium in anticoagulant solutions of the three brands, achieved by the replacement of the 22 mmol/L eluent with the 20 mmol/L eluent. The chromatogram in the rear, marked as Previous B and obtained with the preceding method [19], is presented for comparison. For the modified method, reduction in the sodium peak width and diminishing of the tailing effect are evident.

In Table 2 we present the within-day repeatability of retention times, *t*_r_, and the peak area of the sodium peak of composite anticoagulant samples of blood collection tubes of the three brands. The 95% confidence intervals of retention times were 0.01 min. The 95% confidence intervals of peak area ranged between 2 × 10^6^ and 6 × 10^6^ AU.

The within-day repeatability of fundamental chromatographic parameters of citrate anticoagulant and sodium ion is adequate for development of the methodology for reliable anion–cation balance evaluation.

### 2.2. Ion Exchange Chromatography—Reproducibility of Calibration Model

After confirming the linearity of the dependence of peak area (*y*) on mass concentration of citrate and sodium (*x*), we evaluated within-laboratory reproducibility of the five-point calibration model. The LINEST function of MS Excel was used to obtain regression parameters of the calibration model *y* = *a* × *x* + *b*. Other parameters appearing in Table 3 and Table 4 are standard uncertainty of the slope (*s_a_*), standard uncertainty of the intercept (*s_b_*), standard error of estimate (*s_y_*_/*x*_,) and coefficient of determination *R*^2^.

Mass concentrations of citrate ranged from 6.9 to 34.4 mg/L. The coefficient of determination confirms that the model explains between 99.68 and 99.94% of measurements of the peak area.

Table 4 confirms the within-laboratory reproducibility of regression parameters of calibration line equations for determination of sodium. Mass concentrations of calibration solutions ranged from 246 to 370 mg/L. The coefficient of determination explains between 99.71 and 99.99% of measurements of the peak area.

By confirming the validity of calibration models for citrate and sodium, their quantification became possible. Evaluation of precision, standard uncertainty of interpolation, and accuracy of interpolated concentrations followed.

### 2.3. Ion Exchange Chromatography—Precision, Standard Uncertainty of Interpolation, and Accuracy of Interpolated Concentration

Table 5 provides insight into the precision and accuracy of citrate determination at three concentration levels. Relative standard uncertainty of interpolation of concentration *x*_0_ from the calibration line equation is denoted by *s_x_*_0_/*x*_0_ and evaluated by applying Equation (2). The symbols $x_{i}$, *y*_0_, $\bar{x}$, and $\bar{y}$, not explained previously, stand for the concentrations of calibration solutions, the peak area, and the coordinates of the centroid of the calibration model, respectively. The symbol *m* denotes the number of calibration points and equaled 5. The symbol *n* indicates the number of the sample solution injections.

(2) $$s_{x_{0}}=\frac{s_{y/x}}{a}\sqrt{\frac{1}{m}+\frac{1}{n}+\frac{{\left(y_{0}-\bar{y}\right)}^{2}}{a^{2}\sum {\left(x_{i}-\bar{x}\right)}^{2}}}$$

The bias, *B*, as a measure of the result accuracy, is calculated from Equation (3), where *τ* designates the true or expected value.

(3) $$B=\bar{x}-\tau \;$$

Equation (4) defines the relative percent bias, *B*_r_(%).

(4) $$B_{r}(\%)=100\times B/\tau$$

The 95% confidence intervals of citrate amount concentration ranged between 6 × 10^−4^ and 2 × 10^−3^ mmol/L.

Table 6 confirms accuracy and precision of sodium determination tested on the sodium chloride and trisodium citrate model. The 95% confidence intervals of the sodium amount concentration ranged between 0.01 and 1 mmol/L.

Method performance parameters confirm that the ion chromatographic methods are fit for the purpose of accurately determining citrate and sodium concentration. Consequently, reliable results of the tube examinations and anion–cation balance evaluations can be expected.

### 2.4. Dye-Dilution Method

**Figure 6:**
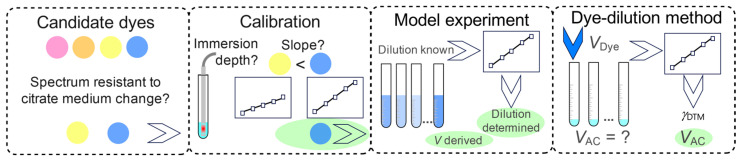
Dye-dilution method development scheme.

In Figure 6 the dye-dilution method development strategy is outlined.

The four candidate dyes were Erythrosine, Sunset Yellow, Tartrazine, and Erioglaucine. The last two qualified for further examinations. Their spectra were robust to changes in the composition of the citrate medium. The calibration experiment confirmed the higher method sensitivity of Erioglaucine compared to Tartrazine (Table 7). Consequently, Erioglaucine was selected for development of the dye-dilution method.

Regression parameters of Series 0 to 3 together with a preceding series (Table 7) prove the reproducibility of the calibration model, explaining between 99.56 and 99.97% of measurements. Erioglaucine calibration standard concentrations ranged from 2.5 to 5.5 mg/L.

As the proof-of-concept, the volume prediction capability test of the dye-dilution method was performed. The dye dilutions modeled the dye concentrations as would have been achieved with the dye-dilution method performed on the tubes with the 150, 180, 200, or 220 μL anticoagulant volumes. The dye mass concentration, *γ*_DTM_, interpolated from a calibration model equation, together with the dye working solution mass concentration, *γ*_D_, and the dye volume, *V*_D_, define the corresponding anticoagulant volume, as stated in Equation (5). The determined *V*_AC_ values were statistically evaluated and compared against the expected values (Table 8).

(5) $$V_{AC}=V_{D}\left(\frac{\gamma_{D}}{\gamma_{DTM}}-1\right)$$

The two-sided t-test, by proving no significant difference between the estimated and expected volume, for any of the volumes, confirmed the suitability of the dye-dilution method for the determination of anticoagulant volume.

Equation (5) indicates three uncertainty contributions to the *V*_AC_ standard uncertainty. The performance characteristics of the 1 mL Brand Transferpette^®^ S adjustable-volume pipette define the standard uncertainty of *V*_D_. Accuracy and precision data provided by the producer for three different volumes were described with the second-order polynomial function to derive the values corresponding to *V*_D_. To transform the volume accuracy data into standard uncertainty, a triangular distribution was presumed. The *γ*_D_ appearing in Equation (5) is further defined by the factor of dilution and the stock dye solution concentration. Regarding the preparation procedure, the uncertainty contribution of the latter can be considered negligible. The uncertainty of the factor of dilution is affected by the measurements of volumes with the 5 mL Brand Transferpette^®^ S adjustable-volume pipette. The same methodology as with the 1 mL pipette was applied to assign accuracy and precision values to volume measurements, which differ from those included in the producer’s specifications. The uncertainty contribution of the *γ*_DTM_ corresponds to *s_x_*_0_, defined by Equation (2). The standard uncertainties were combined and the importance of individual contributions evaluated by a spreadsheet method. The standard uncertainty of interpolation of *γ*_DTM_ from the calibration line equation (*s_x_*_0_) was recognized as the major contributor and the standard uncertainty of *V*_D_ as the minor contributor. The combined relative standard uncertainty of anticoagulant volume determination was 0.015.

### 2.5. Tube Characteristic Evaluation by Developed Methodology

As Table 9 reveals, the pH values determined in blood collection tubes of the three brands after the addition of pre-boiled Milli-Q water in a volume equal to the nominal draw volume are all around 6.0. The citrate anticoagulant of all three brands is buffered, although only producer C declares so.

Average anticoagulant volumes and their variances, determined with the dye-dilution method in the tubes of the three brands, are summarized in Table 10. The lowest average anticoagulant volume of 177.8 μL was observed for tube C, whereas the average volumes of A and A(nr) were close to 190 μL. The mean volume difference proved statistically significant.

Concentrations of the major ions, sodium and citrate, which are essential for the judgement of the anion–cation balance, are given in Table 11 together with their relative standard uncertainties of interpolation, $s_{x_{0}}/x_{0}$. Their concentrations were determined in parallel in the tubes of the three brands after the addition of Milli-Q water in a volume corresponding to the nominal draw volume.

Anionic impurity amount concentrations, determined in composite samples of anticoagulant solutions of the tubes of different brands and lots, are summarized in Table 12. Concentrations relate to the tube-nominal solution volume of 2 mL. Limits of detection expressed in μmol/L were 6.2 for acetate, 3.6 for formate, 2.5 for chloride, 1.6 for nitrite, 2.2 for sulfate, 1.5 for oxalate, 2.7 for bromide, and 2.4 for nitrate. They were obtained by applying the methodology in Ref. [46].

Regarding the declarations of chemical purity, the anticoagulant solution can be considered the source of chloride, oxalate and sulfate. We cannot judge if the sulfur-containing substances, which can leach from a tube stopper [7], can be related to sulfate. For the sources of other impurities, we do not have an explanation. Regarding their concentrations, they are unlikely to affect the results of blood tests.

Within each of the three series, six blood collection tubes were evaluated, two of each brand. For the anionic–cationic composition evaluations, the model solution of trisodium citrate served as a quality control indicator.

The percent anionic–cationic balance ACB(%) is shown in Equation (6):

(6) $$ACB\left(\%\right)=\frac{\sum c_{E-}-\sum c_{E+}}{\sum c_{E-}+\sum c_{E+}}\times 100,$$

which compares the difference between the total anionic and total cationic equivalent concentrations, in the nominator, to the total ionic equivalent concentration, in the denominator, serves as a criterion of the ionic analysis completeness and quality. A mismatch below 5% is considered a confirmation. *c*_E−_ or *c*_E+_ stands for equivalent concentrations of individual anions or cations. The equivalent concentration, in contrast to the amount concentration of an ion, relates to the charge of the ion, e.g., the citrate anion equivalent concentration is three times its amount concentration, but the sodium ion equivalent concentration equals the amount concentration. Therefore, due to the charge neutrality of the solution, the total anionic and total cationic equivalent concentrations would have ideally matched completely if there were no uncertainty contributions to the results of analyses.

The mean ACB(%) of 3.08% (*n* = 9) of the trisodium citrate model solution, by being lower than 5%, confirms the overall quality of ion chromatographic analyses. Neither anionic nor cationic contaminants are present in concentrations that would importantly contribute to the anionic–cationic balance. The between-series comparability of the ACB(%) values of the trisodium citrate model solution, tested with a single factor ANOVA, proves the method reproducibility. Consequently, the blood collection tubes can be reliably evaluated, and results from different series can be considered a homogeneous group.

## 3. Discussion

### 3.1. pH of Anticoagulant Solution

All pH values, measured immediately after the pre-boiled Milli-Q water in a volume corresponding to the nominal draw volume was introduced into the tubes A, A(nr), and C, by not being far off pH 6 (Table 9), confirm similarity between the brands. Reliability of measurements was confirmed with a laboratory-prepared phosphate reference buffer. The pH of 6.80, at 24 °C, was close to the reference pH values of 6.865, as reported by Bates, for 25 °C [47]. The unbuffered sodium citrate model solution exhibited the pH of 8.63. A similar pH was expected for the tubes A and A(nr), since the anticoagulant was declared to be trisodium citrate, but turned out not to be so. Consequently, a difference in the absorption spectra of the anticoagulant solution of the tubes A and C cannot be attributed to the unbuffered and buffered trisodium citrate. If the reason for the difference is the buffer, the method of preparation should differ. Evaluation of the proportion of the amount of sodium to the amount of citrate can reveal this.

### 3.2. Anionic–Cationic Composition

Proportions of the amount of sodium to the amount of citrate of the buffered 105 mmol/L and 129 mmol/L anticoagulant solutions of pH 6.1, derived from the description of the preparation in the patent [48], which comprised the addition of trisodium citrate and citric acid, were 2.40 and 2.51, respectively.

For the proportions derived from the results of Table 11, the single factor ANOVA proved no significant differences between the mean sodium to citrate amount proportions of 2.47, 2.43, and 2.48 of the tubes A, A(nr), and C. On the other hand, the three means significantly differed from the mean of the trisodium citrate model solution. The values confirm that the anticoagulant solutions in the tubes, which after the ten-fold dilution all have a pH between 5.9 and 6.1, were prepared from trisodium citrate and citric acid, similarly as described in the patent. An approach involving the addition of, e.g., HEPES, as suggested to the laboratories during the COVID crisis, or of any other auxiliary buffering substance, proved unlikely. Consequently, the distortion effect on the citrate spectra as observed for tube C and to a lower extent for tube A [18] does not originate from the buffer preparation method.

### 3.3. Determination of Anticoagulant Solution Volume

Since the liquid is not delivered but already contained in a tube, the gravimetric approach was not suitable for volume evaluations. Differential weighing, comprising emptying, cleaning, and drying the tube, would have been required, and the density of the anticoagulant solution should have been known. For the 200 µL volume, Dong et al. confirmed the comparability of the spectrometric and gravimetric calibration procedure [49].

The fiber optic Dip Probe Coupler attached to the Varian Cary 50 UV-Vis spectrometer proved suitable for measurements in low volumes. We observed that the differences in the wall thickness of blood collection tubes of different producers influenced the liquid level at equal volumes. We confirmed that the total solution volume of 500 µL enables the probe to dip adequately no matter the tube brand. Since it was previously observed that not all tubes are water-tight [18], we set a dye volume addition to 400 µL to allow for anticoagulant volume determination down to half of the nominal value, namely 100 µL. Regarding conformity, this limit seems reasonable, since by assuming the correct anticoagulant amount and draw volume, a dilution factor is already changed from ten-fold to nineteen-fold, and the anticoagulant concentration is increased from 10.9 to 11.47 mmol/L. Blood, after being sucked into such a tube, would come into contact with the citrate anticoagulant solution of a concentration of 218 mmol/L, which is two-fold nominal. Quantitative evaluations beyond these limits can be considered of no practical interest.

Tartrazine and Erioglaucine appeared in the literature in drug delivery studies, even though not in equal medium, but in 0.9% sodium chloride, for which the absorption maxima of 430 and 630 nm were reported [50]. Our observations in 36.33 mmol/L citrate medium were 427 nm and 629 nm, respectively, for Tartrazine and Erioglaucine as the prospective dye candidates.

Because it is well known from the early dye-dilution studies that an unmatched dye medium leads to erroneous results [51], we set the objective that the citrate medium had to be corrected for if not present. Since we confirmed that the tubes of all three brands contain the buffered citrate (Table 9), the pH adjustment of the citrate medium was justifiable. We confirmed that 200 µL of trisodium citrate solution, with concentration of 109 mmol/L and an amount of 21.8 µmol, requires an equal volume of 0.1000 mol/L standard HCl solution to ensure that after the 10-fold dilution the solution reaches a pH of 6.0.

Since the line slope calibration of Erioglaucine was approximately 2.75 times higher that of Tartrazine, both in the citrate medium with the adjusted pH, the former was selected for the dye-dilution method, as the higher method sensitivity promises better anticoagulant volume differentiations.

The accuracy and precision of the volume predictions were confirmed with the volume prediction capability test (Table 8). The accuracy of volume predictions in the range between 150 μL and 220 μL proved comparable. The method has been verified to be reproducible. The lowest between-series variability was observed for the target volume of 200 μL.

Because a single-factor ANOVA confirmed insignificant differences between the predicted anticoagulant volume means of the three experimental series for the blood collection tubes of the three brands, which were evaluated in parallel with the method performance tests, the results of each brand were considered a homogeneous group for the between-brands evaluations (Table 10).

The mean anticoagulant volumes of A of 189.8 μL and A(nr) of 191.7 μL proved comparable. The mean anticoagulant volume of C of 177.8 μL differed at the 0.05 significance level from that of A or A(nr), and at the 0.01 significance level it differed from that of A(nr). The *t*-test confirmed that the mean anticoagulant volumes of all three brands were significantly lower than the nominal anticoagulant volume of 200 μL, since the *p*-values of A, C, and A(nr) were 1.9 × 10^−4^, 1.6 × 10^−3^, and 4.6 × 10^−4^. It must be mentioned that tube C was evaluated only 27 days before the expiration date, but tubes A and A(nr) were evaluated 181 days before the expiration date. In tube C, an approximately 10 times higher variance of the anticoagulant volume was observed if compared to A and A(nr) (Table 10).

The relative percent bias of the anticoagulant volume of the tubes A, C and A(nr) was −5.1, −11.1, and −4.2%, respectively. All the values are negative, as reasonably expected, due to a potential water loss over time, but they differ less from the nominal volumes than previously considered [18].

### 3.4. Blood Collection Tubes’ Overall Quality Evaluation

The dye-dilution method for the anticoagulant volume evaluation (*V*_AC_), together with the chromatographic method for the determination of the total citrate amount concentrations in the tubes after adding the Milli-Q water in a volume equal to the nominal draw volume of 1800 µL (*c*_Draw_), enabled the calculation of the anticoagulant concentration in a tube before dilution, *c*_AC_, by applying Equation (7):

(7) $$c_{AC}=\frac{c_{Draw}\times (V_{AC}+1800)}{V_{AC}}{=c}_{Draw}\times f_{AC\_dil}$$

*f*_AC_dil_ defines a factor of dilution of the anticoagulant solution with the nominal draw volume. It is theoretically expected to be equal to 10.

From *V*_AC_ and *c*_AC_ the anticoagulant amount, *n*_AC_, can be calculated.

In Table 13, an overall blood collection tube quality comparison is provided. In addition to the already introduced parameters, the amount concentrations of potassium, magnesium, and calcium are given, namely *c*_K_, *c*_Mg_ and *c*_Ca_. Their concentrations below 38.4, 206, and 3.74 µmol/L, respectively, were not quantified.

The comparison of the results reveals that even though *V*_AC_, *f*_AC_, and *c*_AC_ of tube C exhibit the highest *B*_r_(%), the effects are in different directions. Consequently, *n*_AC_ in the tubes and *c*_Draw_ are the most accurate, with *B*_r_(%) of −0.5% and 0.6%, respectively. Even though *V*_AC_ of tubes A and A(nr) is better preserved over time, the anticoagulant amount demonstrates a positive bias. The conclusion that the anticoagulant of tube A is too high, previously derived from the indirect method of *V*_AC_ determination, was confirmed [18].

On the other hand, the cationic contamination of tube C is the most profound. It must be noted that we confirmed a low-level contamination with calcium for all the tubes. Since contamination of 0.006 mmol/L was also confirmed for the sodium citrate model solution, the source is probably the chemical and not the tube. Contrastingly, tubes A and A(nr) are contaminated with anionic contaminants to a higher extent than tube C, as Table 12 revealed. Acetate, formate, chloride, nitrite, sulfate, oxalate, bromide, and nitrate were all determined in tube A. The peak profiles of A and A(nr) reflect some other unidentified impurities.

### 3.5. Implications and Limitations

By developing a dye-dilution method for a direct determination of *V*_AC_, we enable a reliable determination of *c*_AC_, and quantification of *n*_AC_ in individual tubes. Consequently, a comprehensive insight into tube characteristics is possible.

A dye-dilution method is low cost and easily implemented in medical laboratories to control *V*_AC_, as an essential tube lifecycle parameter. Titter-plate readers can be used instead of the immersive spectrometric probe. The only necessary adaptation is that the aliquot of the resulting dye solution should be transferred from the tube to a titter-plate.

The objectives presented in Figure 2 were mainly achieved, but among the anions, which we identified, are several light-absorbing species. Their influence on the citrate anticoagulant absorption spectra of the molecular absorption spectrometric method for the preanalytical quality evaluation of tubes [18] needs to be further examined. On the other hand, the chromatographic profiles of the impurities were not fully explained, and all the peaks were not recognized. It is not possible to assume the effect of the identified light-absorbing contaminants on the citrate absorption spectrum in HCl medium. Further experiments are necessary. Some summative effect is likely, but we doubt that the distortion in the spectrum shape of the tube C anticoagulant can be explained by them. The buffering method also does not appear to be its cause. The origin and identity of this impurity remain unresolved.

Anionic impurities, which we detected, provide some feedback to the producers. Regarding the concentrations, we doubt that they can influence the results of blood tests. Relating the impurities to their sources is demanding. Merck certificates of impurities in chemicals, namely in trisodium citrate dihydrate and citric acid, state chloride, oxalate, and sulfate among the impurities. Consequently, the origin of these ions can be attributed to anticoagulant solutions. Sulfur and sulfur-containing vulcanization accelerators were confirmed to leach from tube stoppers [7]. We cannot judge if they can be related to sulfate. The origin of other anions is unclear.

It should be noted that tube evaluation using a water model cannot replace tube comparison using blood samples. The first evaluates tubes’ inherent characteristics, and the second their fitness for purpose when implemented in the daily routine of medical laboratories. The two approaches are complementary, irreplaceable, and incomparable, since each of them has its own dedication and provides a different insight. For tubes’ inherent characteristics, the standard considers deviations up to 10% from the nominal values to be acceptable and to not alter the results of laboratory tests [13].

## 4. Materials and Methods

### 4.1. Trisodium Citrate, Purified Water, and Evacuated Blood Collection Tubes

All the chemicals were of analytical reagent grade. Trisodium citrate dihydrate C_6_H_5_Na_3_O_7_ × 2H_2_O (*M* = 294.10 g/mol, 0.99 ≤ *w* ≤ 1.01), CAS: 6132-04-3, Sigma-Aldrich, St. Louis, Darmstadt, Germany, was used to prepare a stock solution with a concentration of 1.7 g/L. Five citrate calibration solutions with concentrations ranging from 6.9 to 34.4 mg/L were prepared from it.

Trisodium citrate dihydrate C_6_H_5_Na_3_O_7_ × 2H_2_O (*M* = 294.10 g/mol, *w* ≥ 0.99), CAS: 6131-04-3, Sigma-Aldrich, Belgium was used to prepare model solutions. More details on other chemicals are given concerning dedicated procedures.

Deionized water, additionally purified through the Milli-Q system (Millipore, Billerica, MA, USA), was used to prepare all solutions—Milli-Q water in continuation.

The evacuated blood collection tubes were obtained from Slovenian local dealers. The letters assigned to them were the same as used previously. The letter A stands for Vacuette^®^, Greiner BIO-ONE, Kremsmünster, Austria; B for Vacutube, LT Burnik d.o.o., Komenda, Slovenia; and C for BD Vacutainer^®^, BD, Plymouth, UK. More details are summarized in Table 14.

### 4.2. Ion Chromatography

#### 4.2.1. Determination of Anions with Ion Exchange Chromatography

The ICS-5000 Ion Chromatograph (Thermo Fisher Scientific Inc., Waltham, MA, USA), consisting of the Eluent Generator Cartridge with hydroxide (KOH), Isocratic Pump, IonPac AS11-HC (4 × 250 mm) column, ADRS 600 (4 mm) suppressor connected in a closed mode, and the Electrochemical detector with a conductometric cell were used for the determination of anions. The eluent flow rate was 1.5 mL/min and the injection volume was 30 µL. For the determination of citrate in the blood collection tubes, after the addition of Milli-Q water in the volume corresponding to the nominal draw volume, the eluent concentration of 35 mmol/L was used. Anionic impurities were determined in composite samples by using the 25 mmol/L eluent.

A composite sample was prepared from three tubes of the same kind and the same lot. The content of each tube was quantitatively transferred into an A-class 20 mL volumetric flask with four successive additions of 1.5 mL of Milli-Q water. After each addition the tube was plugged and inverted several times, and the content was transferred into the flask through a funnel. The potential residuals of citrate were afterwards washed out of the funnel into a volumetric flask, and the volume of solution was made up to the mark with Milli-Q water. The tubes A, A(nr), C, A_old, B_old and C_old were evaluated.

Chemicals for impurities determinations were sodium sulfate, Na_2_SO_4_ (*M* = 142.04 g/mol, *w* ≥ 0.99), Supelco, Darmstadt, Germany; sodium acetate, CH_3_COONa (*M* = 82.03 g/mol, *w* ≥ 0.99), Supelco, Darmstadt, Germany; sodium formate, HCOONa (*M* = 68.01 g/mol, *w* ≥ 0.99), Kemika, Zagreb, Croatia; sodium nitrite, NaNO_2_ (*M* = 69.00 g/mol, *w* ≥ 0.99), MERCK, Darmstadt, Germany; potassium bromide, KBr (*M* = 119.01 g/mol, *w* ≥ 0.995), MERCK, Darmstadt, Germany; sodium nitrate, NaNO_3_ (*M* = 84.99 g/mol), Alkaloid, Skopje, Macedonia; disodium oxalate, (NaCOO)_2_ (*M* = 134.00 g/mol, *w* = 0.995), PanReac AppliChem, Darmstadt, Germany; and sodium chloride, NaCl (*M* = 58.44 g/mol, *w ≥* 0.995 or 0.990 ≤ *w* ≤ 1.005 when dried), EMSURE, Merck, Denmark. Sodium chloride for the determination of chloride was dried at 500 °C for two hours. Other chemicals were dried at 105 °C for two hours. Eight multi-ion calibration solutions comprising acetate, formate, chloride, nitrite, oxalate, bromide, nitrate, and sulfate were prepared; the concentrations of all ions in the calibration solution were equal, and the levels in the consecutive solutions were 0.1, 0.2, 0.5, 0.8, 1.5, 2.3, 3.1 and 5.1 mg/L, respectively.

#### 4.2.2. Determination of Cations with Ion Exchange Chromatography

The DX-500 Ion Chromatograph (Dionex Corporation, Sunnyvale, CA, USA), consisting of the GP-40 Gradient Pump and the ED-40 Electrochemical detector with a conductometric cell was used for the determination of cations. Other components were a 50 µL injection loop, IonPac CS12A (4 × 250 mm) column, IonPac CG12A (4 × 250 mm) guard column, and CSRS 300 (4 mm) suppressor in closed mode of operation. The current was set to 50 mA. The eluent was 20 mmol/L methanesulfonic acid, CH_3_SO_3_H (*M* = 96.11 g/mol, w ≥ 0.990, *ρ* = 1.481 g/cm^3^ at 25 °C, CAS: 75-75-2, Sigma Aldrich, Steinheim, Germany), and flow rate was 1 mL/min.

Potassium chloride, KCl (*M* = 74.55 g/mol, 0.990 ≤ *w* ≤ 1.005, *ρ* = 2 g/cm^3^), Fluka, Germany, and sodium chloride, NaCl (*M* = 58.44 g/mol, *w* ≥ 0.995 or 0.990 ≤ *w* ≤ 1.005 when dried), EMSURE, Merck, Denmark, were dried at 300 °C for two hours. Five potassium ion calibration solutions, prepared from the intermediate solution with a concentration of 75 mg/L, had concentrations ranging from 0.75 to 3.75 mg/L. Five sodium ion calibration solutions, prepared from the stock solution with a concentration of 5.0 g/L, had concentrations ranging from 250 to 450 mg/L.

Magnesium standard solution, *γ*(Mg^2+^) 1000 mg/L (traceable to SRM from NIST, Mg(NO_3_)_2_ in HNO_3_ 0.5 mol/L, *ρ* = 1.016 g/cm^3^, Merck KGaA, Darmstadt, Germany) was used to prepare five magnesium ion calibration solutions with concentrations ranging from 2.5 to 4.5 mg/L. Calcium standard solution *γ*(Ca^2+^) 1000 mg/L (traceable to SRM from NIST, Ca(NO_3_)_2_ in HNO_3_ 0.5 mol/L, *ρ* = 1.014 g/cm^3^, Merck KGaA, Darmstadt, Germany) was used to prepare an intermediate standard solution with a concentration of 30 mg/L. Five calcium ion calibration solutions with concentrations ranging from 0.075 to 0.375 mg/L were prepared from it.

### 4.3. Determination of pH

For pH measurements the SevenCompact™ S220 pH/Ion meter (Mettler-Toledo GmbH, Greifensee, Switzerland) and a pH Sensor InLab^®^ Micro (Mettler-Toledo GmbH, Greifensee, Switzerland) combined pH electrode were used. Three-point calibration was performed with the NIST traceable calibration buffers of pH 4.00 ± 0.02 (25 °C), pH 7.00 ± 0.02 (25 °C), and pH 10.00 ± 0.02 (25 °C) (Metrohm Ltd., Herisau, Switzerland). Ambient temperature was 24 °C.

A control reference phosphate buffer was prepared with boiled Milli-Q water from potassium dihydrogen phosphate, KH_2_PO_4_ ((*M* = 136.09 g/mol, *w* ≥ 99.5%) Merck, Darmstadt, Germany) and disodium hydrogen phosphate, Na_2_HPO_4_ ((*M* = 141.96 g/mol, *w* ≥ 99%) POCH S.A., Gliwice, Poland). The chemicals were dried at 105 °C for two hours. Buffer concentration was 25.01 mmol/L and 25.00 mmol/L for KH_2_PO_4_ and Na_2_HPO_4_, respectively.

### 4.4. Spectrometric Determination of Anticoagulant Volume Accuracy

For direct spectrometric measurements in blood collection tubes, the fiber optic absorbance measuring Dip Probe Coupler (02-101593-00, VARIAN, made in Australia, non-patient equipment, 31-1027, EL02036518) was attached to the Varian Cary 50 UV-Vis spectrometer, Agilent Technologies, Santa Clara, CA, USA, ZDA. The spectrometer settings for spectra acquisition were a scan rate of 300.00 nm/min, a data interval of 0.50 nm, averaging time of 0.1 s, dual beam mode, and baseline correction on.

To weigh dyes, a Balance XPR2U (Mettler Toledo, Greifensee, Switzerland, d = 0.1 μg) was used.

Tartrazine, C_16_H_9_N_4_Na_3_O_9_S_2_ (*M* = 534.36 g/mol, CAS: 1934-21-0, Sigma-Aldrich, Toluca, Mexico) stock solution was prepared in a 25 mL, class A volumetric flask from 17.7886 mg of the chemical.

A stock solution of Erioglaucine, disodium salt, C_37_H_34_Na_2_N_2_O_9_S_3_ (*M* = 792.86 g/mol, CAS: 3844-45-9, Sigma-Aldrich, Jiangsu, China) was prepared in a 500 mL, class A volumetric flask, from 12.7286 mg of the chemical.

Spectra of Tartrazine and Erioglaucine solutions were acquired in the wavelength ranges 300–600 nm and 500–800 nm, respectively, against the buffered citrate blank. For anticoagulant volume determination, the absorbances were read at the absorption maxima of Tartrazine and Erioglaucine, which were 427 and 629 nm.

Seven calibration solutions with equidistant dye mass concentrations ranging from 2.5 to 5.5 mg/L were prepared from the Erioglaucine stock dye solution. The 109 mmol/L trisodium citrate solution and 0.1 mol/L standard HCl solution were added to match the composition as expected for the tubes. For the dye-dilution method, the stock solution was diluted 4.25-fold to obtain the working solution of the mass concentration of 5.990 mg/L. The solution was added into each blood collection tube in a volume of 400 μL, and the content was well mixed to achieve uniform composition.

### 4.5. Sample Size

For each experiment, the number of reptations of measurements, the number of calibration levels, and the number of tubes were optimized by considering the implications for the quality of results regarding resource consumption and time limitations.

## 5. Conclusions

A comprehensive methodology comprising anionic and cationic chromatography, a direct dye-dilution method for anticoagulant volume determination, and pH measurements revealed that the anticoagulant of tubes A and C is buffered. Tube C exhibits the highest anticoagulant amount and concentration accuracy at dilution with the nominal draw volume. Acetate, formate, chloride, nitrite, sulfate, oxalate, bromide, and nitrate impurities were determined in anticoagulant solutions, all in tube A, and some additional impurities were unable to be identified. Tube C exhibited the highest contamination with cations. Calcium was confirmed in low concentrations in all the tubes and in the trisodium citrate model solution. Its origin is probably the chemical and not the tube or the stopper. The proportion of the sodium to citrate amount was very similar for the anticoagulant solutions of all the tube brands, and characteristic of the preparation of buffer solution from trisodium citrate and citric acid. Any other auxiliary chemical does not seem to be involved, and the buffering method cannot explain the distortion of the absorption spectrum of tube C, which we observed in the spectrometric method of quality evaluation [18]. The potential influence of the UV-light-absorbing anionic contaminants still needs to be further examined and the unidentified impurity causing the spectrum distortion revealed.

## Figures and Tables

**Table 1:** Within-day repeatability (*n* = 5) of citrate retention times (*t*_r_) and peak area measurements of the examined composite blood collection tubes’ anticoagulant solutions; *s* and *s*_r_(%) stand for sample standard deviation and relative percent standard deviation. Peak area is expressed in arbitrary units (AU).

Repetition	1.	2.	3.	4.	5.	$\bar{x}$	*s*	*s*_r_(%) (%)
Tubes	*t*_r_ (min)							
A	4.243	4.220	4.227	4.230	4.230	4.230	0.008	0.2
C	4.163	4.213	4.170	4.217	4.217	4.196	0.03	0.6
A(nr)	4.173	4.183	4.173	4.177	4.193	4.180	0.008	0.2
Tubes	Peak area (AU)							
A	1.4506	1.4455	1.4414	1.4538	1.4509	1.4484	0.0049	0.34
C	1.3729	1.3692	1.3726	1.3729	1.3728	1.3732	0.00099	0.07
A(nr)	1.4595	1.4618	1.4623	1.4598	1.4624	1.4612	0.0014	0.10

**Table 2:** Within-day repeatability (*n* = 3) of sodium retention times (*t*_r_) and peak area measurements of examined composite blood collection tube anticoagulant solutions; *s* and *s*_r_(%) stand for standard deviation and relative percent standard deviation. Peak area is expressed in arbitrary units (AU).

Repetition	1.	2.	3.	$\bar{x}$	*s*	*s*_r_(%) (%)
	*t*_r_ (min)					
Tube A	5.25	5.24	5.25	5.247	0.006	0.1
Tube C	5.21	5.22	5.22	5.217	0.006	0.1
Tube A(nr)	5.26	5.26	5.27	5.263	0.006	0.1
	Peak area (AU)					
Tube A	2.401 × 10^8^	2.393 × 10^8^	2.407 × 10^8^	2.400 × 10^8^	7.2 × 10^5^	0.30
Tube C	2.237 × 10^8^	2.275 × 10^8^	2.280 × 10^8^	2.264 × 10^8^	2.4 × 10^6^	1.0
Tube A(nr)	2.371 × 10^8^	2.381 × 10^8^	2.387 × 10^8^	2.380 × 10^8^	8.4 × 10^5^	0.35

**Table 3:** Within-laboratory reproducibility of regression parameters (*y* = *a* × *x* + *b*) and their standard uncertainties, standard error of estimate, and coefficient of determination (*s_a_*, *s_b_*, *s_y_*_/*x*_, and *R*^2^) of the five-point calibration (*n* = 5) for citrate determination. A LINEST function of MS Excel was used to obtain regression parameters of the calibration model.

Date	*a*	*s_a_*	*b*	*s_b_*	*s_y_*_/*x*_	*R* ^2^
2 April 2025	0.0639	0.0013	0.1039	0.0290	0.0276	0.9988
3 April 2025	0.0654	0.0017	0.0741	0.0392	0.0374	0.9979
7 April 2025	0.0653	0.0012	0.0969	0.0265	0.0253	0.9990
8 April 2025	0.0640	0.0021	0.1207	0.0476	0.0454	0.9968
9 April 2025	0.0645	0.0017	0.1179	0.0382	0.0364	0.9980
10 April 2025	0.0639	0.0010	0.0448	0.0234	0.0223	0.9992
11 April 2025	0.0652	0.0015	0.0877	0.0349	0.0333	0.9983
15 April 2025	0.0638	0.0011	0.1109	0.0260	0.0248	0.9990
7 May 2025	0.0586	0.0011	0.0778	0.0256	0.0244	0.9989
8 May 2025	0.0600	0.0008	0.0840	0.0184	0.0175	0.9995
9 May 2025	0.0595	0.0009	0.0946	0.0205	0.0195	0.9993
14 May 2025	0.0601	0.0013	0.1073	0.0291	0.0277	0.9987
3 June 2025 *	0.0624	0.0009	0.1184	0.0202	0.0192	0.9994
4 June 2025 *	0.0631	0.0013	0.0967	0.0286	0.0272	0.9988
5 June 2025 *	0.0626	0.0012	0.0961	0.0267	0.0254	0.9990
6 June 2025 *	0.0636	0.0012	0.0796	0.0265	0.0253	0.9990
16 June 2025 *	0.0641	0.0014	0.1099	0.0308	0.0293	0.9987
23 July 2025 *	0.0614	0.0011	0.0991	0.0255	0.0243	0.9990
24 July 2025 *	0.0624	0.0012	0.1046	0.0265	0.0253	0.9990
25 July 2025 *	0.0622	0.0011	0.1056	0.0244	0.0233	0.9991

* Chromatograph was thermostatted to 25 °C.

**Table 4:** Within-laboratory reproducibility of the regression parameters of five-point (*n* = 5) calibration function (*y* = *a* × *x* + *b*) for sodium determination during a two-month period.

Date	*a*	*s_a_*	*b*	*s_b_*	*s_y_*_/*x*_	*R* ^2^
3 June 2025	3.779 × 10^5^	1.174 × 10^4^	−3.723 × 10^6^	4.193 × 10^6^	1.857 × 10^6^	0.9971
4 June 2025	3.785 × 10^5^	1.717 × 10^3^	−1.575 × 10^6^	6.129 × 10^5^	2.714 × 10^5^	0.9999
5 June 2025	3.597 × 10^5^	6.208 × 10^3^	4.023 × 10^6^	2.217 × 10^6^	9.815 × 10^5^	0.9991
23 July 2025	3.550 × 10^5^	2.511 × 10^3^	1.754 × 10^6^	8.966 × 10^5^	3.970 × 10^5^	0.9999
24 July 2025	3.563 × 10^5^	4.198 × 10^3^	1.795 × 10^6^	1.499 × 10^6^	6.637 × 10^5^	0.9996
25 July 2025	3.576 × 10^5^	2.799 × 10^3^	1.287 × 10^6^	9.994 × 10^5^	4.426 × 10^5^	0.9998

**Table 5:** Precision, uncertainty of interpolation, and accuracy of citrate amount concentration determination evaluated at three concentration levels.

Date	*τ* (mmol/L)	Mean (mmol/L) (*n* = 3)	*s* (mmol/L)	*s_x_*_0_/*x*_0_	*B* (mmol/L)	*B*_r_(%) (%)
9 April 2025	0.0655	0.0650	0.0002	0.038	−0.0005	−0.8
16 June 2025	0.1092	0.1108	0.0003	0.016	0.0016	1.4
14 May 2025	0.1525	0.1532	0.0007	0.013	0.0007	0.4
3 June 2025	0.1092	0.1113	0.0007	0.011	0.0021	1.9
25 July 2025	0.1091	0.1108	0.0002	0.013	0.0017	1.6

**Table 6:** Precision, uncertainty of interpolation, and accuracy of determination of sodium amount concentration evaluated on the sodium chloride and trisodium citrate model.

Date	*τ* (mmol/L)	Mean (mmol/L) (*n* = 3)	*s* (mmol/L)	*s_x_*_0_/*x*_0_	*B* (mmol/L)	*B*_r_(%) (%)
3 June 2025 *	15.22	15.17	0.06	0.0012	−0.05	−0.3
4 June 2025 *	15.22	15.16	0.03	0.00018	−0.06	−0.4
5 June 2025 *	15.22	15.23	0.04	0.00069	0.01	0.1
23 July 2025 **	16.36	16.18	0.30	0.00027	−0.18	−1.1
24 July 2025 **	16.36	16.40	0.005	0.00045	0.04	0.2
25 July 2025 **	16.36	16.09	0.46	0.00030	−0.27	−1.7

* NaCl, ** trisodium citrate.

**Table 7:** Between-series reproducibility of the seven-point (*n* = 7) calibration function (*y* = *a* × *x* + *b*) of Tartrazine and Erioglaucine.

Series	*a*	*s_a_*	*b*	*s_b_*	*s_y_*_/*x*_	*R* ^2^
Tartrazine, citrate	0.0462	0.0005	−0.0364	0.0060	0.0035	0.9994
Tartrazine, citrate, pH	0.0437	0.0012	−0.0080	0.0146	0.0085	0.9962
Erioglaucine, citrate	0.1234	0.0035	−0.0233	0.0144	0.0144	0.9960
Erioglaucine, citrate, pH	0.1206	0.0021	0.0076	0.0088	0.0056	0.9984
Series 0	0.1205	0.0010	−0.0106	0.0042	0.0027	0.9997
Series 1	0.1164	0.0035	0.0006	0.0144	0.0092	0.9956
Series 2	0.1179	0.0035	−0.0021	0.0143	0.0092	0.9957
Series 3	0.1134	0.0011	0.0134	0.0046	0.0030	0.9995

**Table 8:** Dye-dilution method anticoagulant volume prediction capability—proof of concept.

Parameter	150 μL	180 μL	200 μL	220 μL
*V*_AC_1_esitmated_ (μL)	157.83	197.05	213.54	229.59
*V*_AC_2_esitmated_ (μL)	163.27	183.77	208.36	217.47
*V*_AC_3_esitmated_ (μL)	146.22	177.92	198.28	216.15
*V*_AC_4_esitmated_ (μL)	−	181.42	198.28	213.54
*n*	3	4	4	4
Mean	155.8	185.0	204.6	219.2
*s*	8.7	8.4	7.6	7.1
*s*_r_(%) (%)	5.6	4.5	3.7	3.3
*B* (µL)	5.77	5.04	4.62	−0.81
*B*_r_(%) (%)	3.85	2.80	2.31	−0.37
*t*_calculated_	1.148	1.206	1.213	0.226
*p*-value	0.37	0.31	0.31	0.83

**Table 9:** pH of anticoagulant solution in blood collection tubes after the addition of 1.8 mL of pre-boiled Milli-Q water.

pH	A	A(nr)	C
Measured	5.948	5.901	6.115
	5.964	5.896	6.125
	5.940	5.891	6.122
	5.900	5.903	6.157
	5.912	5.878	6.134
Mean Standard deviation	5.93	5.89	6.13
	0.03	0.01	0.02

**Table 10:** Results of the ANOVA test for the anticoagulant volume (μL) determined in the blood collection tubes of different brands; the asterisk at a hanging indent indicates which between-groups differences proved statistically significant according to Tukey’s honestly significant difference (HSD) test as a measure.

Tube-Brands	Count	Sum	Average	Variance	Differences				
A	9	1708	189.8	27.4		*			
C	9	1600	177.8	257				*	
A(nr)	9	1725	191.7	23.6		*			

**Table 11:** Concentrations of sodium and citrate determined in parallel in the tubes of the three brands after the addition of Milli-Q water in a volume corresponding to the nominal draw volume of 1.8 mL and relative uncertainties of interpolation of concentrations of the calibration line equations.

Tubes	Sodum Ion, *c*_Na_ (mmol/L)						Citrate, *c*_Draw_ (mmol/L)					
A	27.980	29.040	28.531	28.215	28.743	27.918	11.71	11.65	11.57	11.35	11.15	11.56
A(nr)	28.986	26.366	27.400	28.195	28.591	27.922	12.25	10.74	11.16	11.78	11.46	11.56
C	26.699	27.545	26.617	26.877	26.744	28.748	11.11	10.94	10.71	10.90	10.78	11.37
**Tubes**	$s_{x_{0}}/x_{0}$											
A	4 × 10^−3^	4 × 10^−3^	6 × 10^−3^	6 × 10^−3^	4 × 10^−3^	4 × 10^−3^	0.02	0.02	0.02	0.02	0.01	0.01
A(nr)	4 × 10^−3^	4 × 10^−3^	7 × 10^−3^	6 × 10^−3^	4 × 10^−3^	4 × 10^−3^	0.01	0.02	0.02	0.02	0.01	0.01
C	4 × 10^−3^	4 × 10^−3^	7 × 10^−3^	7 × 10^−3^	5 × 10^−3^	4 × 10^−3^	0.02	0.02	0.02	0.02	0.02	0.01

**Table 12:** Amount concentrations of anionic impurities determined in composite samples prepared from the blood collection tubes of a particular type and expressed in μmol/L.

Tubes	Year *	*c* (C_2_H_3_O_2_^−^) ± *s* (*n* = 3)	*c* (HCOO^−^) ± *s* (*n* = 3)	*c* (Cl^−^) ± *s* (*n* = 3)	*c* (NO_2_^−^) ± *s* (*n* = 3)	*c* (SO_4_^2−^) ± *s* (*n* = 3)	*c* (C_2_O_4_^2−^) ± *s* (*n* = 3)	*c* (Br^−^) ± *s* (*n* = 3)	*c* (NO_3_^−^) ± *s* (*n* = 3)
A_old	2024	56 ± 12	34 ± 2	41 ± 5	76 ± 4	7 ± 2	21 ± 3	31 ± 4	<5.5
C_old	2023	36 ± 7	44 ± 2	48 ± 2	19.0 ± 0.6	5.5 ± 0.4	8.7 ± 0.2	22.4 ± 0.4	−
B_old	2024	−	−	−	−	<3.6	−	67 ± 4	−
C	2025	>173.2	45 ± 4	56.3 ± 0.9	19 ± 3	<3.6	10 ± 1	29 ± 3	−
A	2026	114 ± 7	24 ± 1	31 ± 3	66.9 ± 0.2	<3.6	16.01 ± 0.02	21 ± 1	<5.5
A(nr)	2026	71 ± 3	21.4 ± 0.5	19.62 ± 0.08	56.5 ± 0.3	<3.6	12.0 ± 0.3	17.9 ± 0.1	−

* Tubes expiration year.

**Table 13:** Overall blood collection tubes quality comparison.

Parameter	A	A(nr)	C	*τ*
MEAN *c*_Draw_ (mmol/L)	11.5	11.5	11.0	10.9
*s* (mmol/L)	0.2	0.5	0.2	—
*s*_r_(%) (%)	1.8	4.5	2.2	—
*B*_r_(%) (%)	5.5	5.4	0.6	—
MEAN *V*_AC_ (µL)	190	192	178	200
*s* (µL)	5	5	16	—
*s*_r_(%) (%)	2.8	2.5	9.0	—
*B*_r_(%) (%)	−5.1	−4.2	−11	—
MEAN *f*_AC_dil_ (1)	10.5	10.4	11.1	10
*B*_r_(%) (%)	4.8	3.9	11	
MEAN *c*_AC_ (mmol/L)	121	119	122	109
*s* (mmol/L)	2	5	3	—
*s*_r_(%) (%)	1.8	4.5	2.2	—
*B*_r_(%) (%)	11	9.5	12	—
MEAN *n*_AC_ (µmol)	22.9	22.9	21.7	21.8
*B*_r_(%) (%)	5.0	5.0	−0.5	—
MEAN *c*_K_ (mmol/L)	*	*	0.131 (*n* = 6) **	—
MEAN *c*_Mg_ (mmol/L)	*	*	0.233 (*n* = 4) **	—
MEAN *c*_ca_(mmol/L)	0.007 (*n* = 3) **	0.004 (*n* = 3) **	0.004 (*n* = 1) **	—

* Below the calibration range. ** In parentheses, the number of tubes, out of the total of six, in which the impurity was possible to quantify, is given.

**Table 14:** Characteristics of the examined evacuated citrate or buffered citrate blood collection tubes.

Abbreviation	Anticoagulant *c* (mmol/L)	Expiration Date	Draw Volume (mL)
A_old **	109	4 July 2024	1.8
B_old **	109	31 January 2024	1.8
C_old **	109 *	31 December 2023	1.8
A	109	1 February 2026	1.8
A(nr)	109	1 February 2026	1.8
C	109 *	August 2025	1.8

* Buffered trisodium citrate according to specification. ** Included because they were involved in the previous study [19].

## Data Availability

The original contributions presented in the study are included in the article; further inquiries can be directed to the corresponding author.
